# Volatile Organic Compounds of *Datura stramonium*: Changes in Response to Induced Leaf Damage Between Native and Non-Native Populations

**DOI:** 10.3390/plants15101501

**Published:** 2026-05-14

**Authors:** John Martin Velez-Haro, Sabina Velázquez-Márquez, Juan Vázquez-Martínez, Ken Oyama, Juan Núñez-Farfán

**Affiliations:** 1 Departamento de Ecología Evolutiva, Instituto de Ecología, Universidad Nacional Autónoma de México (UNAM), Mexico City 04510, Mexico; john.velez@ecologia.unam.mx (J.M.V.-H.); svelazquez@ecologia.unam.mx (S.V.-M.); 2 Departamento de Ingeniería Química y Bioquímica, Instituto Tecnológico Superior de Irapuato, TecNM, Carretera Silao-Irapuato Km 12.5, El Copal, Irapuato 36821, Guanajuato, Mexico; juan.vm@irapuato.tecnm.mx; 3 Escuela Nacional de Estudios Superiores Morelia, Universidad Nacional Autónoma de México, Morelia 58190, Michoacán, Mexico; kenoyama@enesmorelia.unam.mx

**Keywords:** *Datura stramonium*, VOCs, constitutive and induced plant defense, native and non-native populations, GLVs and HIPVs, *TPS10* gene

## Abstract

The ecological interaction between plants and herbivores has promoted the evolution of defense and offense characteristics of both parties. Specialized metabolites, including volatile organic compounds (VOCs), constitute a key defensive mechanism of plants, helping to reduce/prevent damage by herbivores and indirectly attracting their natural enemies. However, in the absence of herbivores, as occurs in invaded ranges, natural selection may favor the reduction in costly chemical defenses. Here, we assessed the production of VOCs in both damaged and undamaged leaves of plants of *Datura stramonium* from Mexico (native) and Spain (non-native). The emissions of VOCs were detected and compared, along with the induction extended to neighboring undamaged leaves. A total of 45 VOCs were detected and differences in chemical diversity and concentration between plants of different origin and between damaged and undamaged leaves. Notably, native populations exhibited greater VOCs diversity and higher emission levels than non-native populations, highlighting population-specific differences in both constitutive and induced chemical defenses. Expression analysis of the gene implicated in terpenoid biosynthesis (*DsTPS10*) demonstrated damage-induced upregulation. Gene expression patterns coupled with metabolic profiles suggest a potential defense capability of native populations as compared with non-native populations of recent evolution in the absence of the *D. stramonium*’s coevolved herbivores.

## 1. Introduction

In nature, herbivore insects can act as plant pests, causing significant losses in forest crops [1], or as pollinators, highlighting a co-evolutionary relationship that has existed for millions of years [2]. This co-evolution continuously adapts the defensive traits to play a significant role in ecological systems and along with it, plants have evolved both direct and indirect defenses against herbivory [3]. These include the production of chemical compounds with multiple biological functions, including pollinator attraction and protection against herbivores and pathogens, and other ecological interactions [4,5]. These compounds include a wide variety of primary and secondary (specialized) metabolites, some of which are constitutively expressed, while others are induced in response to an attack, acting directly on herbivores or indirectly by attracting herbivores’ natural enemies [6,7]. In addition, these same specialized metabolites and their precursors are recognized as signaling molecules involved in regulating plant cell differentiation and growth [8]. It has been documented that plant taxonomic groups exhibit a diverse range of chemical defense classes in response to the specific demands of their ecological niches. It has been further postulated that some of these adaptations have emerged through co-evolution with herbivores, contributing to the remarkable diversity of metabolites in plants [2,8,9].

Specialized metabolites are produced by most plants and are one of the most important defense mechanisms against specialist and generalist herbivores [10,11]. Among the metabolites related to defense are the so-called volatile organic compounds (VOCs), small molecules (<400 Da) that function as chemical signals (e.g., allelochemicals) between species and belong to different classes or chemical groups. These can evaporate at “normal” room temperature and the physical–chemical properties that they possess make them ideal candidates for studying signaling among the interactions of different plant organs (root, leaf, or flower) and tritrophic interactions [12,13,14], playing a significant role in the defense of plants against herbivory [11,14]. Examples of VOCs include herbivore-induced plant volatiles (HIPVs) and green leaf volatiles (GLVs) compounds, which can affect herbivores such as aphids and caterpillars, and act as attractants or repellents to natural enemies [14].

GLVs produced via the lipoxygenase pathway typically include (*Z*)-3-hexenal, (*Z*)-3-hexenyl acetate and (*E*)-2-hexenal, which have been widely reported as components of herbivore-induced volatile blends [15]. Volatile signals can also act within a plant, bypassing vascular restrictions, to transmit damage information systemically [16]. In parallel, terpenoids (e.g., monoterpenes such as limonene and sesquiterpenes such as β-caryophyllene) or octanal are distinctive HIPVs that shape tritrophic interactions by attracting natural enemies, modulating herbivore behavior, and priming defense responses. Several induced volatiles contribute to broader “stress-mitigation processes” in plants, linking specialized metabolism to responses beyond herbivory [17,18,19].

*Datura stramonium* (Solanaceae), commonly known as Jimsonweed or Toloache, is native to America and it is widely distributed around the world [20,21]. This plant serves as a model species to explore the dynamics of plant–herbivore interactions and underlying mechanisms, primarily their chemical defenses, and medicinal properties [22,23,24]. In their natural habitat, it is known to attract specialized herbivores such as *Lema daturaphila*, *Trichobaris* spp., *Epitrix* spp., *Sphenarium purpuracens*, and *Manduca* spp. [22,25,26]. However, it should be noted that all parts of the plant are toxic, due to its high content of tropane alkaloids [23], mainly hyoscyamine and hyoscine (scopolamine) [6], which function as potent direct defenses against herbivory [22]. Additionally, to these constitutive chemical defenses, *D. stramonium* produces a wide array of volatile and non-volatile compounds, including terpenes, tannins, steroids, and carotenoids [27,28,29,30,31,32]. Among these, volatile organic compounds (VOCs) play a central role as indirect defenses, mediating ecological interactions by repelling herbivores, attracting natural enemies, and facilitating intra- and inter-plant signaling. Given this dual defensive strategy, combining toxic alkaloids and inducible VOC emissions, *D. stramonium* provides an ideal system to investigate how different defense components may vary across populations. In particular, the emission of VOCs represents the importance of understanding intraspecific variations in the production of molecules to the responsive to environmental conditions and herbivore pressure. These variations are expected to exist between native and non-native populations, as has been documented for other metabolites [22,25,33,34,35].

The aim of this research was to analyze the volatile organic compounds of *D. stramonium* by comparing two populations: one native (from Mexico) and one non-native (from Spain). These populations have been geographically separated for centuries and have adapted to distinct environmental conditions, with the non-native population living in conditions devoid of its natural enemies. We investigated whether the plant’s metabolic response to damage is influenced by its origin and environmental history. Previous studies have demonstrated that plants of *D. stramonium* receive much less damage by herbivores in the non-native habitats in Spain as compared with populations in the native habitat in Mexico [36] and differences in the quantity of atropine and scopolamine have been documented [34]. However, a common garden experiment indicated that specialist herbivores prefer native plants, suggesting local adaptation of herbivores [22]. Despite these advances, no study has directly compared the constitutive and inducible VOC profiles of native and non-native populations, leaving it unknown whether differences in herbivore pressure have resulted in divergent chemical defense strategies. We predict that *D. stramonium* populations from Mexico, due to their geographic origin and greater exposure to herbivore pressure, will exhibit a more diverse metabolic response to foliar damage when compared to plants from Spain. Therefore, this hypothesis assumes that stronger herbivore pressure in the Mexican regions may have driven the evolution of more effective and diverse chemical defenses in these populations to cope with co-evolving herbivores. In contrast, Spanish populations, which experience relatively lower herbivore pressure, are expected to exhibit a less pronounced emission of volatile compounds in response to damage. These findings could provide valuable insights into the ecological and evolutionary dynamics of this widespread plant species.

## 2. Results

### 2.1. VOCs Profile in Datura stramonium

In our study, we investigated the effects of induced damage and population origin (native and non-native) on the production of metabolites in leaves of *Datura stramonium*. Additionally, we evaluated whether this damage triggered a systemic response (SR) in the plant. The applied damage represents a standardized simulation of herbivory, allowing for controlled comparisons between treatments rather than replicating a natural insect attack. Given the importance of understanding the impact of damage on *D. stramonium* and the ecological relevance of plant responses to injured tissue, we focused primarily on metabolites known for their association with defense-related functions against herbivores.

Overall, we detected 45 volatile organic compounds (VOCs) (Tables S1 and S2) in the leaves of *D. stramonium* plants. Of these, 35 compounds were annotated (Table S2) based on mass spectral data and library matches, while 10 compounds remained unidentified, with molecular weights ranging from 88 to 228 Daltons (C5–C14) (Figure S1). Among these metabolites, ten constitutive compounds were shared between both native and non-native populations. In contrast, thirteen inducible compounds were identified (for both populations), and a limited number of compounds were found exclusively in one of the studied population or origin [dodecane; decanal; 1-octanol; sulcatol; (*E*)-2-hexen-1-ol acetate; (3Z)-3-hexenyl propionate (for non-native); and limonene; (*E*,*E*)-2,4-heptadienal; 2-nonanone; epi-camphor (native)].

In addition, three more constitutive and three more inducible compounds were detected in native populations than in non-native populations (Table S3). However, when comparing the systemic response, native populations exhibited the production of 21 additional compounds in damaged plants, indicative of a systemic response to foliar damage (depending on the population and/or full-sib family), whereas non-native populations showed only 18 distinct compounds from their constitutive profile (see details below).

### 2.2. Analysis of Profile and Family Chemicals of VOCs

**Figure 1:**
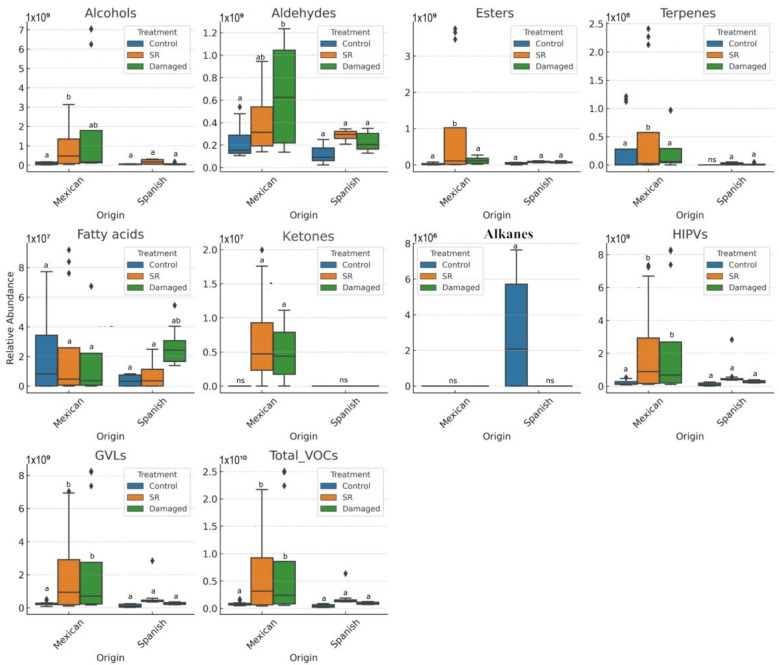
Emission of volatile organic compounds (VOCs) by undamaged (Control), damaged plants (30% mechanical damage), and undamaged leaves of damaged plants (SR, systemic response) in two populations of *Datura stramonium*. Box plots represent the distribution of the data, showing the median (central line), interquartile range (box), and variability (whiskers). Different letters indicate statistically significant differences among groups as determined by Tukey’s HSD test (*p* ≤ 0.05). Black dots represent outliers.

Our study revealed that the proportion of chemical families was very similar between the two populations (origins). However, significant quantitative differences were observed. The most abundant chemical families in leaves were alcohols (22%) and aldehydes (22%) of the total compounds, followed by esters (~11%), GVLs (green leaf volatiles) (14.5%), and HIPVs (herbivore-induced plant volatiles) (13.5%). In contrast, heteroaromatic ketones were present in lower quantities (Figure 1). Two-way analyses of variance (ANOVAs) revealed significant differences in the production of volatile compounds between populations and treatments. After applying false discovery rate (FDR) correction, several of these effects remained statistically significant for several chemical families (*q* ≤ 0.05), particularly for alcohols and aldehydes, while some marginal effects were no longer statistically significant. These results indicate an influence of both origin and induced damage on VOC emissions. For instance, the alcohol family exhibited significant variability, with an F-value of ~7.79 (*p* = 0.0069), suggesting a strong genetic influence on the production of these compounds. Aldehyde emissions differed significantly between origins and these effects remained significant after FDR correction (native vs. non-native; F = 15.06, *p* = 0.0002) and between treatments (control vs. damaged; F = 7.10, *p* = 0.0016), indicating the induction of these compounds in response to foliar damage. It is important to note that chemical families (e.g., aldehydes, alcohols, esters, terpenes) represent structural classifications, whereas GLVs and HIPVs refer to ecological-functional categories. These functional groups may include compounds belonging to different chemical families; therefore, both classifications are complementary and were analyzed separately to distinguish structural from defense-related ecological patterns.

In contrast, the ester family showed a marginal effect of origin (native vs. non-native; F = 3.82, *p* = 0.0549). Alkanes demonstrated a significant induction in non-native populations subjected to simulated damage, with an F-value of 10.27 (*p* = 0.0021), suggesting a defensive-specific response or communication strategy in these populations. Additionally, monoterpenes and esters, although exhibiting less pronounced induction (monoterpenes: F = 0.81, *p* = 0.4490; esters: F = 3.97, *p* = 0.0234), also displayed some variability (see Figure 1, Table 1). The post hoc Tukey honestly significant difference (HSD) test confirmed that Spanish plants emitted significantly higher amounts of monoterpenes and sesquiterpenes compared to Mexican plants, suggesting population-specific variation in chemical defense responses (terpene-based). Interestingly, the interaction between origin and treatment did not consistently show significant effects (*p* ≥ 0.05) across the chemical families analyzed, indicating that genetic variability associated with origin did not uniformly influence the response to induced mechanical damage (Table 2). Significant differences between origins were observed in the emissions of aldehydes, monoterpenes, ketones, and alkanes. Similarly, differences were detected in the emissions of aldehydes, esters, sesquiterpenes, ketones, and alkanes when comparing between treatments.

### 2.3. VOCs Profile and Semi-Quantitative Analysis of Plants with and Without Damage in Mexican and Spanish Populations

In this study on the semi-quantification (relative abundance) of total volatile organic compounds (VOCs) in *Datura stramonium*, we conducted a two-way ANOVA and found that geographic origin accounts for approximately 14.32% of the total variation in VOC concentrations (F = 9.45, *p*-value: 0.003), indicating statistically significant differences between Mexican and Spanish populations, which remained significant after FDR correction (*q* ≤ 0.05). Similarly, the treatment factor contributed to around 10.37% of the observed variation (F = 3.42, *p*-value: 0.039), suggesting that plants respond differently to various treatments for simulated damage. However, the interaction between origin and treatment did not show significant differences (F = 1.98, *p* = 0.146), with only 6.01% of the variation in VOC emissions attributable to the interaction of origin and treatment (cf. Table 2).

**Figure 2:**
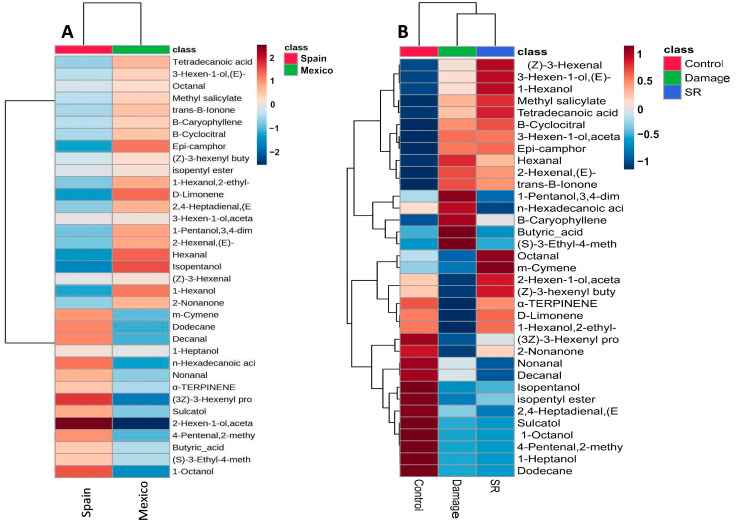
Heat map analysis of relative abundance of 35 leaf metabolites in *Datura stramonium* between origins (native and non-native) of plants (**A**), and treatments (**B**) (control, damaged, and SR). Rows correspond to metabolites, columns to samples grouped by origin/treatment. Color variations from blue to red represent the lowest to highest metabolite abundances, respectively. This analysis used MetaboloAnalyst 6.0 implemented in R (version 4.4.1), Pearson correlation for distance measurement, and Ward’s clustering algorithm.

The main differences found in plants without damage was the decrease in diversity and concentration of metabolites; for example, we found shared molecules in the chemical profile of both populations, but in the Mexican populations the most abundant quantified compounds were epi-camphor, 2-nonanone, (*E*)-2-hexanal, hexanal, isopentanol, limonene, 1-hexanol; whereas for the Spanish populations were (*E*)-2-hexen-1-ol acetate, (3*Z*)-3-hexenyl propionate, 1-octanol, m-cymene, sulcatol, dodecane, decanal. In the heat map, there is a visible increase in the (*E*)-2-hexen-1-ol acetate concentration, which is 2-fold higher on a logarithmic scale (Figure 2). In addition to the differences between origins, the heat map also reveals treatment specific patterns with damaged leaves showing a higher richness and abundance of compounds as hexanal, (*E*)-2-hexenal, (*E*)-3-hexen-1-ol, and terpenoids, while systemic leaves (SR) showed intermediate values between damaged and control leaves, consistent with a systemic induction response (Figure 2B), whereas compounds that including limonene, β-caryophyllene, methyl salicylate, and α-terpinene showed higher emission specifically under certain treatments. Control plants maintained the lowest abundances of VOCs, confirming that most compounds are induced rather than constitutive. These patterns support the hypothesis that foliar damage activates both local and systemic metabolic responses in *D. stramonium*.

### 2.4. Emission Changes of Constitutive and Inducible Compounds in Native and Non-Native Populations of Datura stramonium

We analyzed both constitutive (CVC) and inducible (IVC) volatile compounds emissions in native and non-native populations of *Datura stramonium* (see Table S3). Compounds detected exclusively in both damaged leaves and adjacent healthy leaves, but absent in control plants, were classified as part of an induced systemic response. This pattern indicates that certain volatiles are emitted beyond the directly injured tissue. The presence or absence of these compounds varied by family and population of origin; some families showed a clear systemic emission profile, while others did not.

Furthermore, compounds detected exclusively in control plants and absent in both damaged and adjacent healthy leaves (systemic response) were not classified as part of a systemic response (according to the plant family studied) (Table S2). Instead, their absence could reflect suppression or downregulation following damage, possibly as part of a trade-off in metabolic allocation toward other defense-related volatiles [25].

We observed that in CVC, the populations do not share 19 compounds (eleven native, and eight non-native, respectively), while IVC has thirteen in common (eight native, and five non-native); these results depend on the specific full-sib families used for each population. The relative semi-quantification of analysis of CVC revealed that, on average, plants from the native range emit 1.73-fold higher CVC emission than the non-native (Figure S3A). ANOVA tests indicated significant differences in the levels of constitutive and inducible expression among native and non-native populations of *D. stramonium*. No significant effect of treatments on constitutive compounds was observed (F = 0.95, *p* = 0.3919). In the case of IVC, significant differences were detected (F = 10.18, *p* = 0.0022), showing that native populations emitted 5.73 times more IVC than non-native populations, indicating a clear variance between groups. Additionally, a significant effect was observed on the levels of induced compounds (F = 3.38, *p* = 0.004) (Table 3). The Tukey HSD test highlighted an average difference of −0.3921 in emission levels, which was statistically significant (*p* = 0.002) with a confidence interval of −0.6359 to −0.1484. This finding suggests that origin plays a pivotal role in the emission of inducible volatile compounds, with native populations generally exhibiting higher emissions than non-native counterparts.

### 2.5. Analysis of Alpha Diversity Indices

The richness of metabolites emitted in the leaves of *Datura stramonium* ranged from 10 to 25 compounds in the four populations analyzed (Table 4). In all cases, damaged plants exhibited higher richness than control plants, except in the Valdeflores (Spain) population. The Shannon and Simpson indices showed the same pattern, indicating that foliar damage increased both the number and evenness of emitted metabolites. In contrast, the systemic response did not appear to have a significant impact on metabolite diversity in any of the populations.

Each population exhibited a unique diversity of compounds in response to treatments. The Valdeflores control population displayed the highest compound diversity, whereas the Ticumán showed the greatest diversity when exposed to damage, and the Zubia exhibited the lowest diversity. While richness was consistent across populations, evenness varied, suggesting that certain compounds were more abundant in specific populations, families, and treatments than others. Intrapopulation analysis revealed that Ticumán had the highest diversity and abundance of compounds in the leaves, followed by Zubia, Teotihuacán, and Valdeflores.

### 2.6. Principal Component Analysis of VOCS in Mexican and Spanish Populations

**Figure 3:**
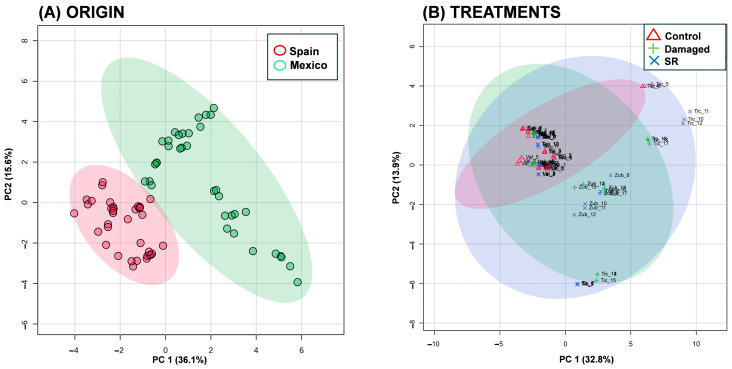
Principal Component Analysis (PCA) of volatile organic compounds (VOCs) between the origin of populations (native: Mexican; non-native: Spanish) and treatments. (**A**) PCA plot for the grouping of samples by population origin (Mexican and Spanish) of *Datura stramonium*. (**B**) PCA plot for Grouping by treatment (Control, Damaged, and SR), plants in the control (triangle symbols), mechanically damaged (plus symbols), and SR (cross symbol) treatments. Labels indicate the population and treatment. Each point represents a sample; ellipses indicate the 95% confidence region for each group based on a multivariate normal distribution.

An analysis of the 35 VOCs revealed distinct groupings between origins and treatments. Principal Component Analysis (PCA) plots exhibited a two-dimensional score distribution and sample clustering. The combined results of the first two PCAs explained more than 50% of the total variance (PC1: 36.1% and PC2: 15.6%) (Figure 3 and Figure S3B), indicating separate groups depending on origin and treatment. However, when comparing treatments with damage and SR, there was some overlap in the treatment ranges, indicating similarities in compounds, but a slight difference between them.

ANOVA analysis indicates that both population (F = 8.31, *p* = 0.0036) and damaged (F = 9.32, *p* = 0.0110) have a statistically significant effect on VOC emissions. The LSMeans analysis and the Tukey test reveal significant differences between the four populations, with Ticumán (LSM = 3.287) followed by Zubia (LSM = 2.638), Teotihuacán (LSM = 2.277), and Valdeflores (LSM = 1.656). Regarding the damaged plants treatment, it was observed that it had a significantly higher emission of volatiles compared to the undamaged plants (LSM = 2.081 vs. LSM = 2.643, *p* = 0.0110), suggesting that the emission of volatiles is influenced by both the origin and the treatment.

### 2.7. Multivariate Analysis of VOCs and Specific Volatile Organic Compounds: GLVs and HIPVs

The GLV and HIPV compounds were detected in higher percentages in *Datura stramonium*, representing 13.5% and 14.5%, respectively, in leaves. In a two-factor analysis of variance, the results revealed statistically significant differences in the emission of herbivore-induced plant volatiles (HIPVs) among the *D. stramonium* populations (F = 88.48, *p* ≤ 0.0001). This marked variation between populations suggests a genetic or adaptive basis for the differential VOC emission. Furthermore, the treatment imposed a significant modification in the HIPV profiles (F = 32.61, *p* ≤ 0.0001), indicating a possible influence of environmental stress factors on the production of secondary metabolites. The interaction between the population origin and the applied treatment showed a highly significant effect (F = 59.88, *p* ≤ 0.0001), suggesting that the response to damage is significantly modulated by the genetic origin of the plants (Table S4). This finding is consistent with a differentiated evolutionary response of the native Mexican populations compared to those introduced in Spain, possibly reflecting an adaptation to divergent environmental pressures. The variability within treatments, represented by the residual term, was considerable (SQ = 3.18 × 10^16^), but less than the variability explained by the studied factors, supporting the robustness of the experimental design and data analysis.

**Figure 4:**
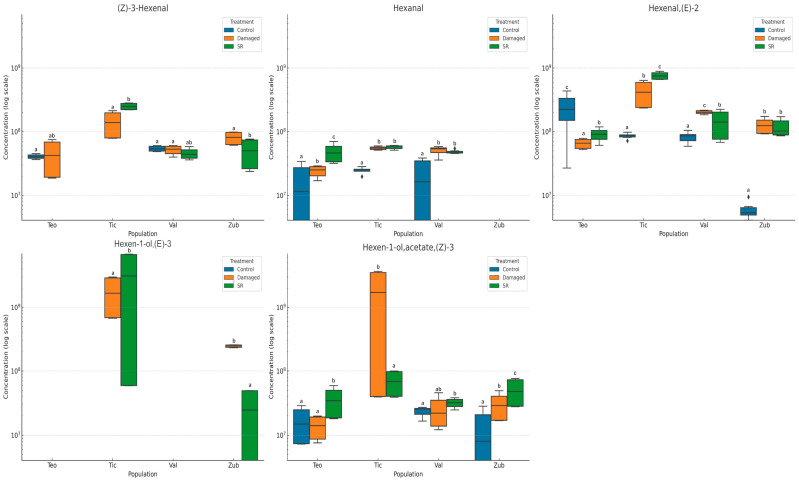
Differences in the emission of herbivore-induced volatiles (HIPVs) between native and non-native *Datura stramonium* plants in different treatments. Box plots show log10-scaled relative abundance of representative HIPVs across populations (Teo = Teotihuacán, Tic = Ticumán, both from Mexico; Val = Valdeflores and Zub = Zubia, both from Spain) and treatments (control, damaged, systemic response [SR]). Panels correspond to individual compounds. Different letters indicate statistically significant differences among groups according to Tukey’s HSD test (*p* ≤ 0.05).

The identified metabolites belong to groups known to be involved in defense (HIPVs), with seventeen found in *D. stramonium*, thirteen of which are common between populations (Figure 4). At the origin level, unique molecules were found in each population (Table S2). In the comparative analysis of each compound within the HIPV groups, a two-factor ANOVA followed by Tukey’s HSD test revealed significant differences (*p* ≤ 0.05) in the emission of HIPVs among populations and treatments. After applying FDR correction, several of these effects remained statistically significant (*q* ≤ 0.05), particularly for compounds such as (*E*)-2-hexenal and related volatiles [as (*E*)-2-hexen-1-ol acetate, and (3*Z*)-3-hexenyl propionate], whereas other compounds did not retain significance [(*Z*)-3-hexenal, (*Z*)-3-hexen-1-ol-acetate, and (*Z*)-3-hexenyl butyrate] after adjustment, indicating that some effects were marginal. However, Mexican populations exhibited higher concentrations of total HIPVs and individual compounds such as hexanal and (*E*)-2-hexenal than Spanish populations.

When analyzing the interaction of origin vs. treatment, six compounds in the control group showed very low to intermediate concentrations (Figure 4). However, in the damaged and SR treatments, a significant increase in the production of (*Z)*-3-hexenal, hexanal and (*E*)-3-Hexen-1-ol is observed. The ANOVA test for the case of HIPVs, it is observed that for most compounds, there are significant differences between origins and treatments. The values in the tables indicate that the production of hexanal is primarily influenced by the treatment, with a substantial amount of explained variance, followed by (*Z)*-3-hexenal and (*E*)-2-hexenal (Figure S4). By analyzing the local and SR at 18 h in both induced and constitutive leaves of the damaged plants, a clear trend in the response to the stimulus was noticed, with the levels of some defense-related metabolites increasing.

At the population level, we found that Ticumán had the highest diversity and abundance of compounds in leaves, followed by Zubia, Teotihuacán, and Valdeflores (Figure 4). From the global analysis, we found molecules involved in plant defense: (*Z*)-3-hexenal, (*E*)-2-hexenal, β-ciclocytral, trans-β-ionone, and a possible relationship between the synthesis route and the ratio of these molecules. We observed an increase/decrease in these compounds in both Mexican populations, indicating that every population has the same chemotype according to the main component analysis. This finding summarizes the metabolic differences between the Spanish and Mexican populations. MeSA, also known as methyl salicylate, is a crucial molecule in signaling the SR in plants. We observed that this molecule was only detected in two of the studied populations, Ticumán and Zubia. The results show that the concentration of MeSA is higher in Mexican populations and in damaged plants. These results suggest that the production and release of MeSA could be related to the resistance of plants to damage stress.

**Figure 5:**
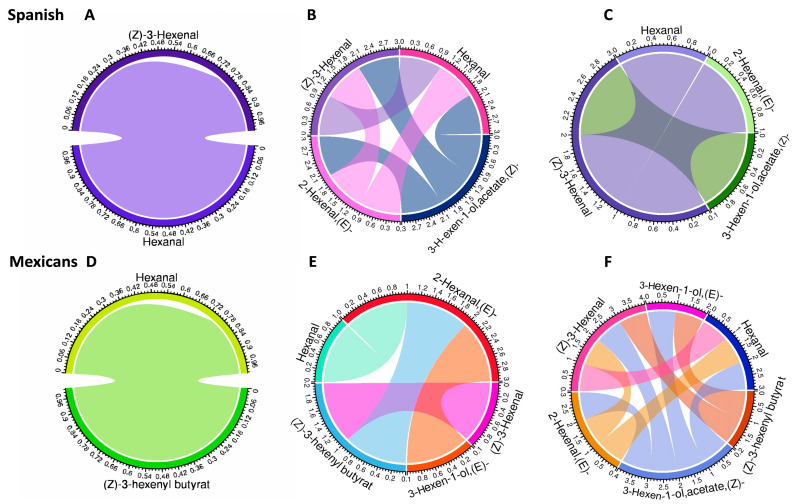
Correlation networks of HIPV metabolites by origin and treatment of *Datura stramonium* plants. Chord-style correlation networks among selected HIPVs were constructed separately for each origin x treatment combination. Panels (**A**,**D**), control plants; (**B**,**E**), Damage plants, and (**C**,**F**) SR plants. The colored lines correspond to positive and negative correlations. Mexican populations showed a greater number of positive and negative interactions in the SR and damage treatments.

Metabolite network analysis showed differences between treatment and origin (Figure 5). The interaction network in Spanish populations, the compounds (*E*)-2-hexanal and (*Z*)-3-hexanal are the main metabolites with a negative relationship with other metabolites, whereas the other metabolites showed positive correlations (the colored lines in Figure 5 correspond to positive and negative correlations). The Mexican populations showed a greater number of positive and negative interactions in the damage and SR treatments compared to the control and shared the greatest number of molecules (except for two of the blue lines that have more connections than the control network).

We tested global differences in leaf VOC compositions using Permutational Multivariate Analysis of Variance (PERMANOVA), with permutations constrained within Family to respect the nested design. Population explained a fraction of the variance (*R*^2^ = 0.255; F = 14.89; *p* = 0.001) and Treatment had a significant effect (*R*^2^ = 0.090; F = 5.27; *p* = 0.001). Because Population is fully nested within Origin, the marginal effect of Origin was not estimable once Population was included.

Tests of multivariate dispersion indicated heterogeneous dispersion among populations (PERMDISP, F = 10.05; *p* = 1.45 × 10^−5^), whereas no dispersion differences were detected among treatments (PERMDISP, *p* = 0.95); thus, population differences reflect both centroid shifts and dispersion, while the treatment effect is robust to dispersion. A sensitivity analysis using Bray–Curtis dissimilarities on row-normalized data yielded consistent conclusions (Population *R*^2^ = 0.245; *p* = 0.001; Treatment *R*^2^ = 0.094; *p* = 0.001; PERMDISP among populations F = 12.21; *p* = 1.76 × 10^−6^).

Hierarchical clustering of samples (Hellinger + Euclidean) recapitulated the PERMANOVA pattern, with samples grouping primarily by Population and showing within-population structure by Treatment (Figure S5A). Clustering of metabolites using 1 − Spearman’s ρ identified coherent modules of co-varying VOCs across samples (Figure S5B), consistent with treatment and population, dependent shifts in VOC blends. The correlation structure among VOCs (Figure S5C) was assessed using pairwise Spearman correlations on Hellinger-transformed intensities. This analysis revealed a structured covariation among metabolites, which remained significant after Benjamini–Hochberg FDR procedure (*q* ≤ 0.05). Significant associations were predominantly positive, forming coherent modules that correspond to the clusters in Figure S5B. The strongest FDR-significant positive association was between (*E*,*E*)-2,4-heptadienal and 2-nonanone (*ρ* = 1.00), indicating near-perfect covariation across samples, and the strongest negative association was between (*Z*)-3-hexenyl butyrate and n-hexadecanoic acid (*ρ* = −0.60), indicating inverse modulation. Non-significant pairs are omitted (white) in the plot for clarity.

### 2.8. TPS10 Gene Expression Analysis

To evaluate the expression level of the *TPS10* gene under simulated damage, we compared the four genotypes (Teo = Teotihuacán, Tic = Ticumán, Val = Valdeflores, and Zub = Zubia) from *Datura stramonium* and the two locations of origin. We identified that the *TPS10* (*DsTPS10*) gene was upregulated at 18 h after damage, mainly in Ticumán (Tic) genotype, with a 5.23-fold change, while in the other genotypes the expression level of the *TPS10* gene does not change significantly (fold change ≈ 1) (Figure S6). In addition to the foregoing, only Tic genotype showed a statistically significant increase compared to other genotypes (*p ≤ 0.05*), suggesting a genotype-specific variation in *TPS10* gene induction.

## 3. Discussion

Plants possess multiple defense mechanisms against herbivory, from constitutive to inducible, both of which contribute to adaptive chemical plasticity. Inducible defenses are important because they reduce metabolic costs by being expressed only under stressful conditions, such as herbivore attack or mechanical damage [37,38], although it is important to recognize that mechanically induced damage does not fully reproduce the complexity of natural herbivore interactions. Moreover, the chemical composition of non-native plants is regarded as a crucial phenotypic trait contributing to metabolite diversity and, therefore, to natural selection [39,40]. In this context, *Datura stramonium* constitutes an excellent model for studying how evolutionary history and environmental context shape chemical defense strategies, especially its specialized metabolites (such as VOCs), which are critical for different responses (defense signaling and ecological interactions) [37,41].

Although there is limited research in our study on the profile of VOCs of *D. stramonium* with respect to induced foliar damage, we demonstrate interpopulation differences in the emission and diversity of VOC compounds between native (Mexico) and non-native (Spain) populations of *D. stramonium*. Native populations exhibited (for the most part) greater richness and emission levels of both constitutive and inducible volatiles. Although our study focused on mechanical damage, this pattern is consistent with previous studies showing that plants exposed to higher herbivore pressure tend to have a greater investment in chemical defenses [42,43]. Several of the VOCs identified in this study have been linked to stress perception signals that help stabilize the reactive oxygen species (ROS) or as a response of plants to herbivory [13,44,45,46]. In particular, several monoterpenes and apocarotenoids detected in this study, such as β-cyclocitral and nonanal, and β-ionone, have been involved in defense and can inhibit germination and growth of plant pathogens, repel herbivores, or attract herbivorous parasitoids (HIPVs) [45,47].

We also detected evidence of a systemic/aerial signaling signature: GLVs, such as hexenyl acetate, that are fast signals and can activate defense gene expression in undamaged tissues and even neighboring plants, reducing the cost of maintaining full defenses at all times (constitutive defenses) [48]. This is consistent with previous reports describing GLVs as rapid signaling molecules involved in the activation of systemic defense [49,50]. In addition, apocarotenoids (β-cyclocitral, trans-β-ionone) and terpenoids (e.g., β-caryophyllene, α-terpinene, limonene) varied by treatment and origin, consistent with their roles in herbivore control and natural enemy attraction. Terpenoids/HIPVs can modulate predator and parasitoid behavior (including attraction to compounds such as DMNT (4,8-dimethyl-1,3,7-nonatriene)/nerolidol and enhanced indirect defense through manipulation of pathways related to the *TPS* gene), and (*E*)-β-caryophyllene can recruit entomopathogenic nematodes to injured roots [19,45,47,51]. Furthermore, apocarotenoids function as regulatory signals, and terpenoids can be transported between tissues (“natural pesticide”), supporting the idea that induced signals propagate beyond wounded tissue and/or natural enemy attraction, and impact whole-plant defense by activating volatiles in intact leaves [15,45,52,53]. The accumulation of β-cyclocitral in the Ticumán and Zubia populations represents a chemical defensive trait that enhances the plant’s ability to withstand herbivore attacks. In *Arabidopsis thaliana*, β-cyclocitral is known to regulate root growth and mediate plant responses to environmental stresses such as oxidative stress and intense light [54].

Certain chemical groups, such as esters and fatty acids, tended to decrease after foliar damage, whereas alcohols and aldehydes increased after damage, indicating a response of compounds involved in the direct and indirect plant defense. The qualitative and quantitative variation observed between populations, treatments and genotypes with damage, suggests that specialized metabolites are phenotypically plastic and are determined by the selective environment, generating a metabolic cost and an effect of the difference between populations and genotype of the plant [8,22,55,56,57,58]; generally, the native plants produced a higher chemical diversity and more abundant constitutive and induced compounds. These patterns align with the eco-evolutionary hypothesis that historical herbivore pressure in their natural distribution area may favor a stronger chemical defense, whereas non-native populations may diverge in allocation while retaining a subset of distinctive compounds [36,59,60,61].

While the observed differences in VOC profiles between Mexican and Spanish populations are consistent with the hypothesis that historical differences in herbivore pressure may have contributed to the divergence in defensive chemistry, our data do not allow us to exclude alternative explanations. Some of the observed variation could also reflect other alternative hypotheses, such as founder events, stochastic differentiation, or indirect responses to other selective factors that differ between native and non-native distribution areas, such as climatic conditions, soil characteristics, biotic interactions, or those associated with the phyllosphere or rhizosphere, and human management [62,63,64]. Therefore, the present results should be interpreted as evidence of population-level divergence in VOC emission patterns under simulated damage. To discern among these alternative hypotheses will require further studies such as common-garden experiments in multiple introduced populations, reciprocal comparisons between environments, and the integration of genomic (which are few), ecological, and biotic interaction data under natural herbivory. In addition, it is important to acknowledge the technical limitations associated with VOCs analysis. These compounds are highly dynamic; they are continuously emitted, transported, and degraded, making them difficult to capture. VOC measurements using gas chromatography–mass spectrometry (GC–MS) are restricted to specific sampling windows, which can lead to overlooking temporal variations in emission patterns. These methodological limitations should be considered when interpreting differences in VOC profiles between populations. Hence, the importance of having controlled and standardized experiments.

Together, these results suggest that not only the presence or absence of specific metabolites, but also their relative proportions under the effects of mechanically induced stress in plant leaves and in relation to herbivore pressure, balance constitutive allocation with the economy of inducible responses, a pattern predicted by the cost/benefit theory of defense and the relationship between chemistry and function in specialized metabolism [41,48,57,62,65]. Multivariate analyses reinforce these inferences, separating samples by origin and treatment, while hierarchical clustering and correlation structure revealed coherent modules of metabolites. These results show that induced and systemic VOCs signaling differ from individual compounds to family-level but also across population-level modules, which determine the magnitude of these responses. Correlation structure is consistent and suggests a possible pathway for the regulation of VOCs (e.g., GLVs via the lipoxygenase pathway; terpenoids via TPSs; apocarotenoids from carotenoid cleavage). The prevalence of positive correlations suggests coordination within pathways, whereas the main negative correlation points to allocation trade-offs between chemical groups. In addition, it has been reported that the family of terpene synthases is involved in the synthesis of VOCs, and the observed population-specific variation in these compounds suggests that the evolution of HIPVs profiles in *D. stramonium* is shaped by local adaptation, possibly modulated by genotypic regulation of terpene biosynthesis.

Recent research has demonstrated that the presence of herbivores raises the transcript levels of multiple terpene synthase (TPS) genes and other enzymes involved in the metabolism of terpenoids [66,67,68]. In this sense, the overexpression of the *DsTPS10* gene detected in the Ticuman population suggests a link between transcriptional regulation and terpenoid production, supporting the hypothesis that gene duplication events in *D. stramonium* TPS families may have enhanced its metabolic versatility [46,69,70]. One of the strongest arguments to consider, the induction of the *DsTPS10* gene following foliar damage, together with the accumulation of terpenoid compounds, indicates a possible local and systemic defensive response against herbivores and pathogens, as well as its ability to accumulate in damaged and distal tissues of the plant [46,47,66].

In addition, evidence from Solanaceae species indicates that terpene synthase (TPS) gene families play a central role in shaping chemical profiles through transcriptional regulation and gene diversification. Variation in TPS gene expression has been associated with differences in terpene blends that influence ecological interactions, including herbivore deterrence and attraction of natural enemies [69]. In this context, the differential expression of *DsTPS10* observed in our study may contribute to population-level variation in VOC emissions between native and non-native populations. Such variation in volatile profiles could have ecological consequences in the invaded range. Changes in VOC emissions may alter how non-native populations interact with local herbivores and their natural enemies, potentially affecting indirect defense mechanisms and plant–insect communication. This could lead to shifts in ecological interactions compared to the native range, and may contribute, at least in part, to the success of *D. stramonium* as an invasive species. However, further studies under natural conditions are needed to directly evaluate these ecological outcomes. It is important to note that gene expression was evaluated in a single full-sib family per population, which limits inference at the population level. Additionally, no direct correlation analysis was performed between *DsTPS10* expression and terpene emission; therefore, the relationship between gene expression and VOCs profiles should be interpreted as an association rather than a demonstrated causal mechanism. Altogether, our results provide further evidence that differences in VOC emissions, multivariate metabolic structure, and upregulation of the *DsTPS10* gene between populations of *D. stramonium* to simulated foliar damage are consistent with long-term evolutionary responses under different ecological contexts, including, among others, different herbivore regimes.

These findings could link metabolic, ecological, and genetic perspectives, demonstrating how variation in both constitutive and inducible chemical responses underpins local adaptation and invasion success, contributes to intraspecific differentiation in chemical defense strategies, remaining within the limits of a mechanically induced stress. Together, these results highlight how population-level variation in defense signaling (as VOCs) may shape ecological interactions and adaptive potential across native and non-native ranges. It is important to note that the experimental design is based on mechanically induced damage under controlled conditions and therefore does not fully replicate the complexity of natural herbivore–plant interactions. Consequently, this study is limited to mechanical damage and does not replicate herbivory. Our interpretations are therefore restricted to plant responses to simulated damage, and further studies incorporating real herbivores are needed to validate these patterns under natural ecological conditions.

## 4. Materials and Methods

### 4.1. Plant Populations of Datura stramonium

We selected and analyzed four populations of *Datura stramonium*, two native plants from the populations of Ticumán and Teotihuacán (Mexico), and two non-native plants derived from Valdeflores and La Zubia populations (Spain) (Table S5). For all experiments and analyses of leaves, we selected two full-sib families from each population. We chose six plants of similar size per family. These populations were selected based on their phenotypic traits, alkaloid production, tolerance to herbivory under natural conditions, and their relationship to their geographic origins [70,71]. In addition, to reinforce this study, we also considered the field assessments of the proportion of leaf area removed by herbivores, which indicated marked differences between populations of both origins (ranges). The average proportion of leaf area damaged by herbivores in 1999 was 0.109 and 0.365 for Ticumán and Teotihuacán, respectively [72]. In contrast, a very low proportion of leaf damage was observed at Valdeflores and La Zubia in 2011 (0.02 and 0.034, respectively) [36]. An experimental study, growing all four populations in a Mexican locality (Teotihuacán), detected differences in the average (±S.E.) proportion of leaf damage between origins (ranges); the native populations received more damage than the non-native ones (0.208 ± 0.010 and 0.173 ± 0.011, respectively) [22], supporting consistent differences in herbivore pressure between origins.

### 4.2. Plant Material and Germination Conditions

Fruits were collected from each mother plant (from their natural populations) and were individually bagged and labeled, and seeds were subsequently extracted from each fruit. Seeds of each fruit constitute a full-sib family. The plants of each family used in the experiments are related as full sibs, given that derived from seeds of the same fruit. In the laboratory, to improve germination, seeds were immersed in sterile distilled water at 60 °C for 10 s, followed by eight consecutive 10 min rinses in tap water at 37 °C, from January to February 2020. The seeds were then imbibed for 24 h. After this, we removed the seed coat using a fine-tip tweezer and then settled the seeds in a Petri dish with moistened filter paper [22]. Petri dishes were transferred to a growth chamber with controlled environmental conditions (Conviron, model G30, Winnipeg, MB, Canada), simulating a long day photoperiod, with a 16:8 h of light:dark, temperatures of 35 °C (day) and 27 °C (night), relative humidity in the growth chamber was maintained at approximately ~85%.

After 15 days, when cotyledon leaves were fully expanded, the seedlings were transplanted into individual 2 L pots filled with a 2:1 mixture of sterilized sand and perlite. Plants were kept at constant watering conditions (100 mL per pot/day) and fertilized every two weeks with 20:20:20 Peters^®^ Professional plant nutrient solution (ICL Specialty Fertilizers, Dublin, OH, USA) (20% total nitrogen [4.1% NH_4_^+^, 5.5% NO_3_^−^, 10.4% urea], 20% P_2_O_5_, and 20% K_2_O) at an application rate of 2 g/L. Seedlings were maintained under controlled greenhouse conditions with a long day photoperiod (approximately 16 h light, 8 h dark), day/night temperature of 25 °C/18 °C, and light intensity of 100–200 μmol/m^2^/s. Plants were allocated randomly to benches in the glasshouse and re-randomized again every week to homogenize environmental variance. To avoid unintentional mechanical stimulation, care was taken not to touch the plants; similarly, watering and fertilizing applications were made on the soil, avoiding touching the plants. A separation of 60 cm between plants was maintained throughout the experiment.

### 4.3. Collection of Samples for the Measurement of Volatile Compounds Produced by Datura stramonium

At the onset of flowering, when the plants were about ten weeks in age, sample collection of leaf volatiles was performed. All sampled plants were selected to be of similar size and stage of development, to minimize phenotypic variation that could influence VOC emissions. Three plants of each family were assigned randomly to the control group (no damage) and three to the foliar damage group. Plants in the damage treatment (simulated foliar herbivory via mechanical damage) received 30% of damage in all but one leaf. To determine the amount of damage a given leaf would receive, we measured its leaf length and then obtained the total leaf area by the regression equation, Leaf area = 0.329 × (leaf length)^2^ (N = 120, *r^2^* = 0.987, *p* ≤ 0.001) [73]. Using a cork borer, we made holes in the leaf blade (of fixed diameter) to mimic damage produced by the larvae of the leaf beetle *Lema daturaphila* (Chrysomelidae), the main trophic specialist herbivore of *Datura stramonium* [22]. The number of holes a leaf received was determined based on the inner area of the cork borer and the area of the leaf. Plants of a family were damaged or kept undamaged. At the time of damage application, the plants allocated to the control group of each family were transferred to a contiguous glasshouse room, isolated from damaged plants. Damaged plants were separated according to their family and population. Mechanical damage was applied once per plant prior to VOCs sampling.

Based on established headspace solid–phase microextraction–gas chromatography–mass spectrometry (SPME–GC–MS) methodologies, several previous pilot experiments, and to determine the emission of volatiles and fine-tuning the method (see Supplementary Methods: Preliminary studies), we optimized the sampling protocol to determine VOC emissions, and we collect individually the leaf of the control (undamaged) and damaged plants, 18 h after damage, in a sterilized glass bottle (previously washed and deodorized at 121 °C/15 pounds of pressure for one hour) [74,75,76]. In addition, this interval coincides with the approximate duration of floral anthesis in *D. stramonium* (~18–20 h), a biologically relevant window during which volatile signaling is expected to have ecological significance. Therefore, this interval captures both sustained damage-induced responses and systemic signaling processes (laboratory observations).

In addition, we also evaluated whether the response to the induced damage was local or systemic [systemic response (SR)]; to do this, we took the damaged leaf and the next undamaged leaf [Leaves: The fifth (damaged) and sixth (no damage) leaves (in vivo) were cut from each plant allocated to the damage group]. To avoid errors resulting from mechanical excision, all leaves (control, damaged, and systemic) were cut and processed following the same protocol [77,78,79].

The leaf sample was manually homogenized 60 times inside the respective glass bottle using a glass pestle (previously washed, sterilized, and heat-deodorized) at a crush-hit velocity of one hit/two seconds; this methodology was implemented and standardized. Once the leaf was shredded, the headspace vial was sealed with a thermally deodorized sheet of aluminum foil. The glass bottle with a sealed vial was then placed inside an incubation chamber at 40 °C for 10 min. After the incubation period, the SPME fiber (solid phase microextraction divinylbenzene/carboxen/polydimethylsiloxane (DVB/CAR/PDMS)) 50/30 μm (Supelco Inc., Bellefonte, PA, USA) was introduced through the aluminum seal. We exposed one cm of the fiber to the bottle’s headspace by placing it about 1.5 cm above the tissue surface without touching it and then incubated it at 40 °C for 30 min to allow the absorption of the compounds onto the exposed fiber [74,75,76]. Finally, we immediately desorbed the fiber into the injection port of the gas chromatograph. We followed the same protocol to simultaneously collect all the samples from plants of all groups. Two types of controls were included during VOCs sampling to ensure data reliability. The first consisted of an empty vial exposed to experimental conditions, which served as a procedural blank, and the second control consisted of a glass bottle exposed to the greenhouse environment to detect any volatiles present in the environment (both glass bottles without the plant leaf). All samples were run in a randomized order to avoid potential bias during GC–MS analysis.

### 4.4. Analysis by Means of Gas Chromatography Coupled with Mass Spectrometry of the VOCs

Samples were analyzed by a GC System (Perkin Elmer model Clarus 580, Waltham, MA, USA) coupled to an electron impact ionization mass spectrometer (Perkin Elmer model 560S, Waltham, MA, USA) (EIMS). The injector temperature was 250 °C. The chromatography phase was made in an Elite-5 MS capillary column (30 m × 320 µm × 0.25 µm) (Perkin Elmer, Inc., Waltham, MA, USA) and helium gas (99.999% purity) was used as carrier gas, at a constant flow rate of 1 mL/min. The GC oven program began at an initial temperature of 35 °C, held for 5 min, and then increased at a rate of 10 °C min^−1^ up to 100 °C, held for 5 min, and a second ramp of 15 °C min^−1^ was applied to reach 300 °C, held for 1 min. The transfer line temperature was set at 200 °C. Mass spectra were obtained at 70 eV of electron energy. Measurements were performed in SCAN mode with *m*/*z* range set to 34–450. The ion source temperature was set at 230 °C and operated at 2.9 scans per second. The data obtained by the GC–EIMS were examined with the software TurboMass (Perkin Elmer, Inc., Waltham, MA, USA; version 5.4.2, 2008). The software AMDIS version 2.66 (National Institute of Standards and Technology, Gaithersburg, MD, USA) (http://www.amdis.net/; accessed on 3 October 2025) was used for the determination of the retention time and the extraction of the mass spectrum of each component in the chromatograms. The Mass spectra library software and Database NIST MS Search version 2.0 (National Institute of Standards and Technology, Gaithersburg, MD, USA, 2008) was used for compound annotation. To annotate each compound, we assessed the similarity between the spectra being compared, considering the presence and proportion of the ions. Compound identification was performed by comparing mass spectra with the NIST library, considering match factors ≥85% as acceptable for presumptive identification. In addition, compound identification was evaluating the quality of chromatographic peaks (e.g., peak shape, resolution, and absence of coelution) to ensure signal purity. Consistency of retention time between replicates was also considered and, where possible, compared with published retention indices. Retention time consistency across replicates and, when available, comparison with reported retention indices were also considered. VOCs semi-quantification was performed from chromatographic peak areas obtained by SPME–GC–EIMS analysis. Consequently, the reported values represent semi-quantitative relative abundances (% of total chromatographic area per sample) (see Table S2), which were obtained using the TurboMass software (version 5.4.2.1617; PerkinElmer Inc., Waltham, MA, USA). The area was normalized according to the fresh weight of the plant leaf to obtain the corrected semi-quantitative abundance of each compound [75,76].

### 4.5. Quantitative Reverse Transcription-PCR (RT-qPCR) Analysis

We analyzed the expression of *TPS10* (Terpene Synthase 10), a gene involved in the biosynthesis of terpenes. First, using the published genome of *Datura stramonium* obtained by our group [6,70], we searched for candidate constitutive genes (i.e., “housekeeping”) by *in silico* studies in *D. stramonium* and other Solanaceae species. Our results indicated that *TPS10* is present in many different species and is also present in *D. stramonium* (*DsTPS10*). A previous study conducted by our research group evaluated the role of this gene in *D. stramonium*, including evidence of gene duplication [69]. *In silico* and experimental analyses were performed among several candidate genes to identify suitable references for expression studies. The *EFα* (an elongation factor, *DsEFα*) gene was selected as a constitutive control (although an *Actin* gene was also identified as a strong candidate). It is important to note that the *D. stramonium* genome is still being fully annotated, which limits the availability of well-characterized genes associated with specific metabolic pathways. However, as mentioned previously, our study was conducted using both Spanish (non-native) and Mexican (native) genotypes, the latter of which have been sequenced by our research group. This provides a robust genomic framework to support gene selection and study. Primers were designed based on genomic and transcriptomic sequences obtained from *D. stramonium* genotypes characterized in our laboratory, ensuring sequence-specific amplification. Primer specificity was confirmed by melting curve analysis, showing single amplification peaks, and by inspection of amplification profiles. Amplification efficiency was evaluated using standard curves, with acceptable efficiency values ranging between 90 and 110% and correlation coefficients (R^2^) ≥0.98, and slope values between −3.58 and −3.10, with an optimal slope of −3.32 corresponding to 100% efficiency. Only primer pairs meeting these criteria were used for further analysis. The list of primers used is shown in Table S6. Total RNA was isolated from *D. stramonium*’s leaf tissue, collected at the same time as sampling leaves for VOCs analysis (see above). One full-sib family per population was screened, with three biological replicates per treatment group (control or damaged plants) in the assay. The RNA was isolated with the kit Zymo Research (Quick-RNA MiniPrep, Zymo Research, Irvine, CA, USA), according to the manufacturer’s directions. RNA concentration and quality were measured by the absorbance in a NanodropOneC (Thermo Fisher-Scientific, Madison, WI, USA), and its integrity was determined by electrophoresis in a denaturing agarose gel [65,80]. For quantitative Real Time-PCR (RT-qPCR), 50 ng of total RNA were used per reaction. The validation was performed with the KAPA SYBR FAST One-Step Master Mix (2×) Kit (KAPAByosystems Pty Ltd., Cape Town, South Africa) and was quantified with a Step One Real-time PCR system (Applied Biosystems, Waltham, MA, USA). Each biological replicate was analyzed in triplicate to ensure technical consistency (*n* = 3 × 3). The results obtained were analyzed using the 2^−ΔΔCT^ method [81].

### 4.6. Statistical Analysis

Leaf volatile organic compounds (VOCs) were extracted for all detected VOCs, for *n* = 72 samples (2 origins × 4 populations nested within origin × 2 families per population x 3 biological replicates × 3 treatments). These values were used for all subsequent statistical analyses. Non-detections were treated as zeros prior to transformation. VOCs were grouped both by chemical family (structural classification) and by ecological function (GLVs and HIPVs), following established definitions in plant defense ecology.

Principal component analysis (PCA) was performed on log-transformed relative abundance data to assess patterns of covariation among metabolites, after confirming data normalization and variance homogeneity. Score plots were visualized with Metaboanalyst and ggplot2, and 95% confidence ellipses were generated with stat_ellipse (method = “norm”), which represents the multivariate normal confidence region for each group (Origin or Treatment), the ellipses illustrate the within-group variation. Additionally, diversity analysis of Shannon–Wiener by origin and treatment was conducted. All experiments were performed in triplicate. Data were log-transformed to improve normality and homoscedasticity, which were verified using the Shapiro–Wilk and Levene tests, respectively (*p* ≥ 0.05). Differences between treatments and origins were analyzed using one-way or two-way ANOVA, depending on the experimental question: one-way ANOVA was applied to evaluate treatment effects within each origin, and two-way ANOVA was used to test the interaction between origin and treatment factors under a completely randomized design. Mean comparisons were performed using Tukey’s HSD test (with *p*-values of 0.01 and 0.05). To control for multiple testing in univariate analyses, *p*-values were adjusted using the Benjamini–Hochberg false discovery rate (FDR) procedure. Statistical significance was determined based on FDR-adjusted *p*-values (*q* ≤ 0.05). Because multiple univariate tests were performed across compounds, these analyses should be interpreted as exploratory and considered in conjunction with multivariate results. Thus, inference is based primarily on consistent multivariate patterns rather than on individual compound-level significance alone. The experimental design is hierarchical (family nested within population, and population nested within origin); however, simplified models were used for univariate analyses, and the results were interpreted primarily at the population level.

The sample and metabolite matrix were processed as follows: Hellinger transformation of VOCs intensities (square-root of row-wise proportions) using decostand (method = “Hellinger”). Sensitivity analysis on row-normalized abundances (proportions) with Bray–Curtis dissimilarity. Unless otherwise stated, downstream analyses used Hellinger-transformed data, and to test for global differences in VOC profiles, we used Permutational Multivariate Analysis of Variance (PERMANOVA, 999 permutations) with Euclidean distances on Hellinger-transformed data (α = 0.05). We evaluated homogeneity of multivariate dispersion with PERMDISP, where dispersion differed among groups, and pairwise differences were examined with Tukey’s HSD on distances between group centroids. As a robustness check, we repeated PERMANOVA on Bray–Curtis dissimilarities from row-normalized data; conclusions were consistent. To assess pairwise associations among VOCs, we used Spearman’s rank correlation on the Hellinger-transformed sample for the metabolite matrix and non-detections were set to zero before transformation; VOCs with zero variance were removed, and metabolites detected in fewer than three samples were excluded. Two-sided *p*-values were adjusted for multiple testing with the Benjamini–Hochberg false discovery rate (BH-FDR); significance was declared at *q* < 0.05.

All the statistical analyses were conducted using the R package (R version 4.4.1) [82] and the Metaboanalyst software (Version 6.0) [83]. Analyses used R packages vegan (v. 2.7-3; for PERMANOVA, PERMDISP), factoextra (v. 2.0.0; for visualization), dendextend (v. 1.19.1; for annotation), cluster (v. 2.1.8.2; for silhouette), prcomp (package included in R version) and ggplot2 (v. 4.0.2) both for PCA, and RColorBrewer (v. 1.1-3, for palettes).

## 5. Conclusions

Conducted investigations showed the foliar damage-induced emission of volatile organic compounds by *Datura stramonium* plants differed between native (Mexicans) and non-native (Spain) populations. Native populations exhibited greater richness and emission of constitutive and inducible volatiles, particularly GLVs and HIPVs, in line with studies where herbivores have shaped stronger chemical defenses of native plant populations. Multivariate analyses indicated that the population’s origin and treatment of damage affect the variation in VOCs, while correlation structures among these suggest relationships within biosynthetic pathways. Furthermore, the upregulation of the *DsTPS10* gene, particularly in the native Ticumán population, indicates that terpenoid biosynthesis is transcriptionally activated in response to foliar damage. This is related to the greater emission of terpenoids observed in native plants, reflecting an adaptive characteristic of the inducible chemical defenses of *D. stramonium* in diverse ecological environments. In the context of mechanically induced stress, our results are consistent with divergent adaptive defense strategies between native and non-native populations and provide integrative evidence connecting metabolomic responses (constitutive and inducible) in plant defense, supporting theories of defense evolution and invasion ecology. This work highlights how variation in the regulation of VOCs can underlie phenotypic plasticity and influence a species’ ability to thrive in contrasting environments.

## Figures and Tables

**Table 1:** Two-way ANOVA of the chemical family between origins (native and non-native) and treatments (control, damaged, and systemic response) of *Datura stramonium*.

Source of Variation	d.f.	F-Value	*p*-Value	q-Value	Chemical Family ^a^
Origin	1	7.78	* 0.0068	* 0.0145	Alcohols
Treatment	2	2.59	0.0819	0.1116	Alcohols
Origin × Treatment	2	2.46	0.0928	0.1210	Alcohols
Residual	66				Alcohols
Origin	1	15.06	* 0.0002	* 0.0015	Aldehydes
Treatment	2	7.09	* 0.0016	* 0.0079	Aldehydes
Origin × Treatment	2	3.00	0.0563	0.0804	Aldehydes
Residual	66				Aldehydes
Origin	1	3.82	0.0548	0.0804	Esters
Treatment	2	3.97	* 0.0234	* 0.0428	Esters
Origin × Treatment	2	3.48	* 0.0364	* 0.0458	Esters
Residual	66				Esters
Origin	1	9.69	* 0.0027	* 0.0079	Monoterpenes
Treatment	2	0.81	0.4490	0.4644	Monoterpenes
Origin × Treatment	2	0.73	0.4829	0.4829	Monoterpenes
Residual	66				Monoterpenes
Origin	1	9.53	* 0.0029	* 0.0079	Sesquiterpene
Treatment	2	7.97	* 0.0007	* 0.0042	Sesquiterpene
Origin × Treatment	2	4.87	* 0.0105	* 0.0210	Sesquiterpene
Residual	66				Sesquiterpene
Origin	1	2.53	0.1157	0.1446	Fatty_acids
Treatment	2	1.04	0.3579	0.3834	Fatty_acids
Origin × Treatment	2	2.16	0.1225	0.1470	Fatty_acids
Residual	66				Fatty_acids
Origin	1	24.26	* 5.92 × 10^−6^	* 0.0001	Ketone
Treatment	2	6.59	* 0.0024	* 0.0079	Ketone
Origin × Treatment	2	6.59	* 0.0024	* 0.0079	Ketone
Residual	66				Ketone
Origin	1	10.17	* 0.0021	* 0.0079	Alkane
Treatment	2	10.17	* 0.0001	* 0.0010	Alkane
Origin × Treatment	2	10.17	* 0.0001	* 0.0010	Alkane
Residual	66				Alkane
Origin	1	8.23	* 0.0055	* 0.0126	HIPVs
Treatment	2	3.39	* 0.0395	* 0.0485	HIPVs
Origin × Treatment	2	1.84	0.1664	0.1848	HIPVs
Residual	66				HIPVs
Origin	1	8.78	* 0.0042	* 0.0105	GVLs
Treatment	2	3.34	* 0.0412	0.0501	GVLs
Origin × Treatment	2	1.91	0.1548	0.1786	GVLs
Residual	66				GVLs

^a^ Analysis performed on chemical families of volatile organic compounds in *D. stramonium*. * Asterisks indicate statistically significant effects in the ANOVA (*p* < 0.05) and/or later were adjusted using the Benjamini–Hochberg FDR procedure; statistical significance is based on *q* ≤ 0.05.

**Table 2:** Two-way ANOVA results for total VOC emissions in *Datura stramonium* leaves, testing the effects of geographic origin, treatment, and their interaction.

Source of Variation	d.f.	F-Value	*p*-Value	q-Value
Origin	1	9.45	* 0.0031	* 0.0093
Treatment	2	3.42	* 0.0386	* 0.0493
Origin × Treatment	2	1.98	0.1458	0.1458
Residual	66			

* Asterisks indicate statistically significant effects in the ANOVA (*p* < 0.05) and/or later were adjusted using the Benjamini–Hochberg FDR procedure; statistical significance is based on *q* ≤ 0.05.

**Table 3:** Two-way ANOVA results for constitutive and induced volatile compounds in *Datura stramonium*.

Source of Variation	d.f.	F-Value	*p*-Value	q-Value
Constitutive				
Origin	1	9.23	* 0.0033	* 0.0078
Treatment	2	0.95	0.3919	0.3919
Origin–Treatment Interaction	2	2.93	0.0605	0.0907
Residual	66			
Induced				
Origin	1	10.18	* 0.0022	* 0.0078
Treatment	2	3.38	* 0.0039	* 0.0078
Origin–Treatment Interaction	2	2.02	0.1406	0.1687
Residual	66			

* Asterisks indicate statistically significant effects in the ANOVA (*p* < 0.05) and/or later were adjusted using the Benjamini–Hochberg FDR procedure; statistical significance is based on *q* ≤ 0.05. Post hoc Tukey HSD tests were performed separately to evaluate pairwise differences among treatments.

**Table 4:** Alpha diversity indices of Mexican (Teotihuacán and Ticumán) and Spanish (Valdeflores and Zubia) populations in the leaves by genotypes and treatment.

Population	Treatments	Richness	Shanon	Simpson
Teotihuacán	Control	10	2.458727	0.6425351
	Damaged	12	1.368186	0.8745913
	SR	13	1.851889	0.8833482
Ticumán	Control	18	1.567604	0.8926374
	Damaged	25	2.529541	0.5367506
	SR	25	2.134699	0.7479104
Valdeflores	Control	16	2.465915	0.8940086
	Damaged	12	2.072327	0.819745
	SR	12	2.000437	0.7889543
Zubia	Control	12	2.246606	0.8689923
	Damaged	21	2.605519	0.9011899
	SR	20	1.270067	0.4743188

## Data Availability

The data presented in this study are available from the corresponding author upon reasonable request.

## Supplementary Materials

The following supporting information can be downloaded at: https://www.mdpi.com/article/10.3390/plants15101501/s1, Table S1: VOC metabolites detected in the leaves of *Datura stramonium*’s plants (Native vs. Non-native); Table S2: Relative abundance (% of total chromatographic peak area) of volatile organic compounds identified in *Datura stramonium* leaves (Excel file); Table S3: Constitutive (CVC) and induced (IVC) volatile compounds detected in native and non-native populations of *Datura stramonium*; Table S4: Two-way ANOVA results for the emission of GLVs and HIPVs in *Datura stramonium* leaves by population and treatment; Table S5: Origin and classification (native vs. non-native) of the four *Datura stramonium* populations analyzed; Table S6: Primer sequences used for RT-qPCR analysis of gene expression in *Datura stramonium*; Figure S1: Representative chromatograms of volatile organic compounds (VOCs) collected from *Datura stramonium* leaves and analyzed using GC–EIMS; Figure S2: Emission of constitutive volatile compounds (CVCs) (A); and induced (B) by native and non-native genotypes of *Datura stramonium*; Figure S3: (A) Relative quantification of volatile compounds methyl salicylate, β-cyclocitral, and trans-β-ionone in *Datura stramonium* populations revealed significant differences, with increased emission observed in the damage treatment, followed by the systemic resistance (SR) treatment. (B) Principal Component Analysis (PCA) biplot showing the separation of samples according to Origin and the type of associated metabolites; Figure S4: Differential VOCs Response to leaf damage in *Datura stramonium* populations; Figure S5: Hierarchical clustering and Spearman correlations among VOCs data in *Datura stramonium*. (A) Dendrogram of samples based on Euclidean distances from Hellinger-transformed VOC intensities. (B) Dendrogram of metabolites based on 1 − Spearman’s *ρ* across samples; shaded rectangles indicate the k clusters. (C) Pairwise Spearman correlations of VOCs (Hellinger-transformed), circle color encodes sign (blue = positive, red = negative) and size (diameter); Figure S6: Relative expression profile of *TPS10* of damaged plants of *Datura stramonium* as compared to control plants; Supplementary Methods: Preliminary studies.
