# Supplementary material for: Volatile Organic Compounds of Datura stramonium: Changes in Response to Induced Leaf Damage Between Native and Non-Native Populations
Source: Plants (Basel). 2026 May 14;15(10):1501. doi: 10.3390/plants15101501 (PMC13211237; doi:10.3390/plants15101501)
Supplement: Supplementary file 1 [file plants-15-01501-s001.zip › Supplementary_Materials_JNF.pdf]

Supplementary Materials

Changes in VOCs metabolites in response to induced damage between native and non-native populations of *Datura stramonium*

John Martin Velez-Haro[1,†], Sabina Velázquez-Márquez[1,†], Juan Vázquez-Martínez[2], Ken Oyama[3], and Juan Núñez-Farfán[1,*]

Table S1. VOC metabolites detected in the leaves of *Datura stramonium´*s plants (Native vs. Non-native). (Provided as a separate Excel file).

Table S2. Relative abundance (% of total chromatographic peak area) of volatile organic compounds identified in *Datura stramonium* leaves. (Provided as a separate Excel file).

Table S3. Constitutive (CVC) and induced (IVC) volatile compounds detected in native and non-native populations of *Datura stramonium*. [a]

	Constitutive compounds (CVC)		Inducible compounds (IVC)	
**Index**	**Native**	**No native**	**Native**	**No native**
**1**	Isopentanol	4-Pentenal,2-methyl-	(Z)-3-Hexenal	(Z)-3-Hexenal
**2**	Hexanal	Hexanal	Hexanal	Hexanal
**3**	2-Hexenal,(E)-	2-Hexenal,(E)-	3-Hexen-1-ol,(E)-	3-Hexen-1-ol,(E)-
**4**	1-Hexanol	1-Hexanol	1-Hexanol	1-Pentanol, 3,4-dimethyl-
**5**	1-Pentanol, 3,4-dimethyl-	1-Pentanol, 3,4-dimethyl-	1-Pentanol, 3,4-dimethyl-	[c]Butyric_acid
**6**	3-Hexen-1-ol, acetate, (Z)-	3-Hexen-1-ol, acetate, (Z)-	[b]Butyric_acid	3-Hexen-1-ol, acetate, (Z)-
**7**	Octanal	2-Hexen-1-ol,acetate,(E)-	3-Hexen-1-ol, acetate, (Z)-	Octanal
**8**	m-Cymene	1-Heptanol	[c]1-Heptanol	m-Cymene
**9**	D-Limonene	Sulcatol	2,4-Heptadienal,(E,E)-	(S)-3-Ethyl-4-methylpentanol
**10**	(S)-3-Ethyl-4-methylpentanol	Octanal	Octanal	isopentyl ester
**11**	1-Hexanol,2-ethyl-	(S)-3-Ethyl-4-methylpentanol	D-Limonene	α-Terpinene
**12**	isopentyl ester	(3Z)-3-Hexenyl propionate	(S)-3-Ethyl-4-methylpentanol	Nonanal
**13**	α-Terpinene	1-Octanol	1-Hexanol,2-ethyl-	Methyl-salicylate
**14**	Nonanal	Nonanal	2-Nonanone	[c]Decanal
**15**	(Z)-3-hexenyl butyrate	(Z)-3-hexenyl butyrate	(Z)-3-hexenyl butyrate	B-Cyclocitral
**16**	Methyl-salicylate	Dodecane	Epi-camphor	[b]B-Caryophyllene
**17**	B-Cyclocitral	Decanal	Methyl-salicylate	[b]trans-B-Ionone
**18**	B-Caryophyllene	n-Hexadecanoic acid	[b]B-Cyclocitral	[b]Tetradecanoic acid
**19**	trans-B-Ionone		[b]B-Caryophyllene	
**20**	Tetradecanoic acid		[b]trans-B-Ionone	
**21**	n-Hexadecanoic acid		[b]Tetradecanoic acid	

* Note: [a] Detected depending on the population and/or full-sib family and population analyzed.

[b] Present in both damaged and undamaged leaves of damaged plants.

[c] Present only in undamaged leaves of damaged plants.

Table S4. Two-way ANOVA results for the emission of GLVs and HIPVs in *Datura stramonium* leaves by population and treatment.

Source of Variation	DF	F Value	P Value
Population	3	88.48	0.0001
Treatment	2	32.61	0.0001
Population x Treatment	4	59.88	0.0001
Residual	30		

Table S5. Origin and classification (native vs. non-native) of the four *Datura stramonium* populations analyzed.

Population	Locality	Country	Geographical coordinates (Lat, Long)	Altitude (m a.s.l.)	Genetic Background	Families	Habitat	Treatments	Reference
Teo	Teotihuacan, State of Mexico	Mexico	19°41′33″N, 98°50′38″W	2280	Native	2	Xerophilous scrubland	Control<br>Damaged<br>SR	22
Tic	Ticumán, Morelos	Mexico	18°45′40″N, 99°07′09″W	972	Native	2	Dry tropical forest	Control<br>Damaged<br>SR	22
Zub	La Zubia, Granada	Spain	37°7′47.28″N, 03°35′57.06″W	692	Non-native	2	Crop field	Control<br>Damaged<br>SR	37
Val	Valdeflores, Sevilla	Spain	37°43′2.23″N, 06°18′50.44″W	287	Non-native	2	River bank	Control<br>Damaged<br>SR	37

Table S6. Primer sequences used for RT-qPCR analysis of gene expression in *Datura stramonium*.

Gene Name	Orientation	Sequence (5'-3')	Amplicon length (bp)
DsTPS10	Forward	GCGTCGCTGCAAGTGATATACG	230
	Reverse	CCATGGACTCTCAGATGTGCAGC	
DsEFa	Forward	GCTGAACGTGAGCGTGGTATCACC	239
	Reverse	CCGAGGGTGAAAGCAAGCAAAGC	

*Reference: [71,84]

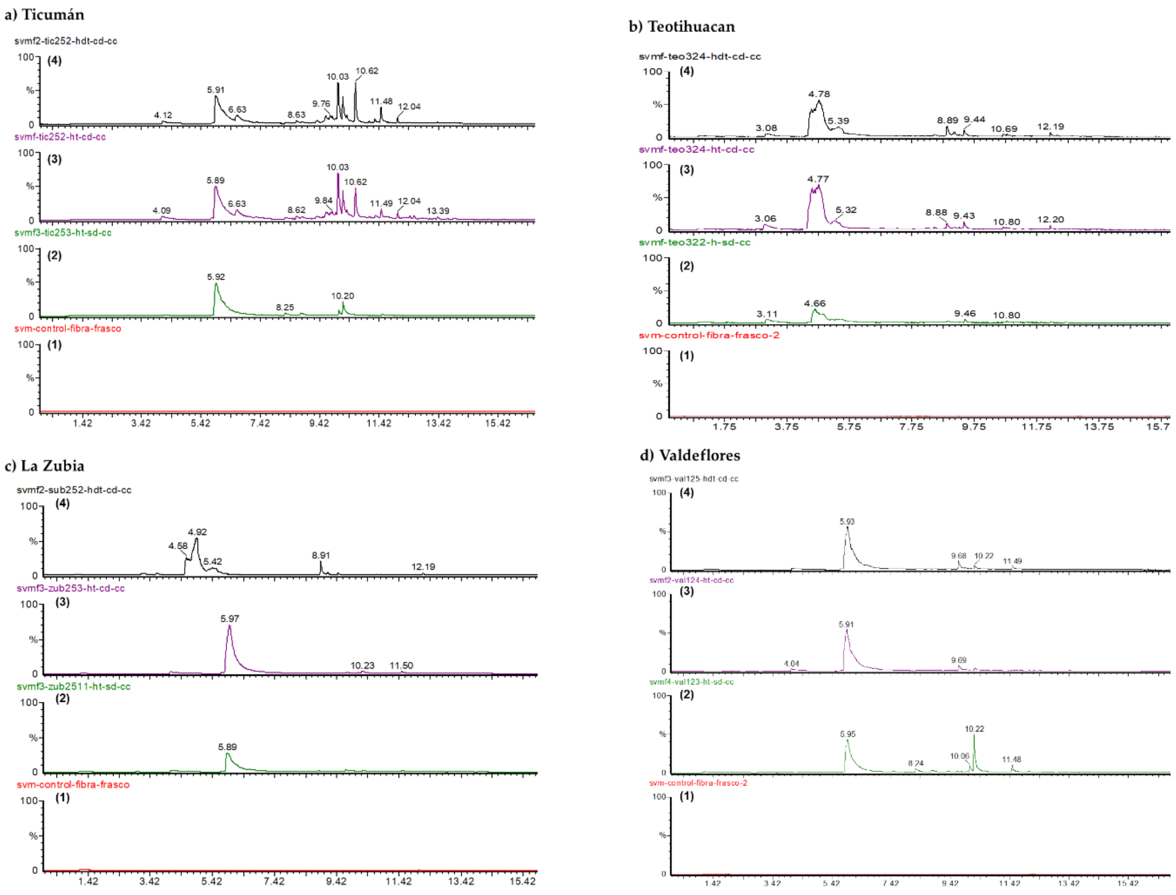

Figure S1. Representative chromatograms of volatile organic compounds (VOCs) collected from *Datura stramonium* leaves and analyzed using GC-EIMS. The figure is divided into four panels corresponding to different genotypes sampled from distinct geographic locations: (a) Ticumán (Tic), (b) Teotihuacan (Teo), (c) La Zubia (Zub), and (d) Valdeflores (Val). Each panel displays four chromatograms (from bottom to top) representing: (1) control, sterilized glass bottle; (2) control leaf from an undamaged plant; (3) undamaged neighboring leaf from a damaged plant (30% foliar damage); and (4) damaged leaf from the damaged plant (30% foliar damage). VOCs were captured using SPME fibers and analyzed 18 hours post-treatment.

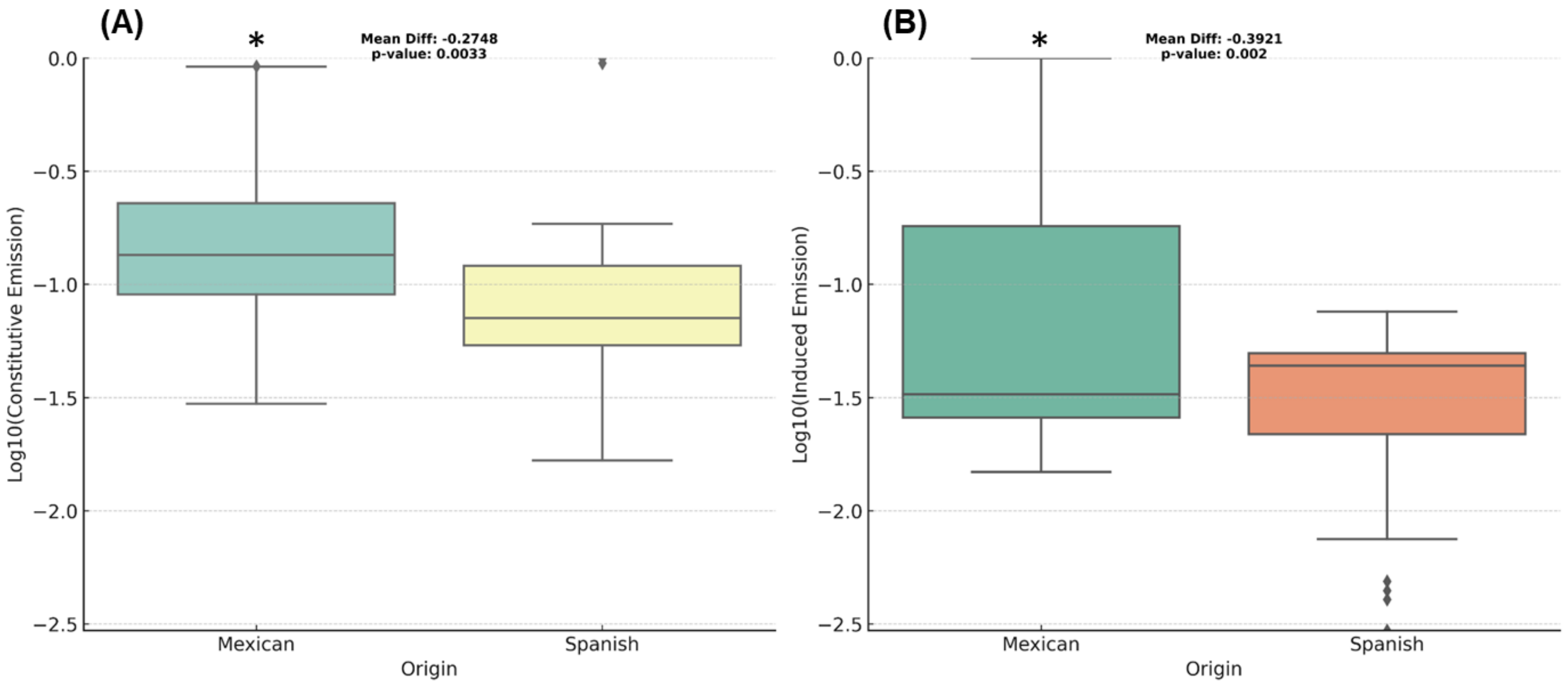

Figure S2. Emission of constitutive volatile compounds (CVC) (A); and induced (B) by native and non-native genotypes of *Datura stramonium*. Asterisks indicate significant differences between origin; $*p < 0.05$.

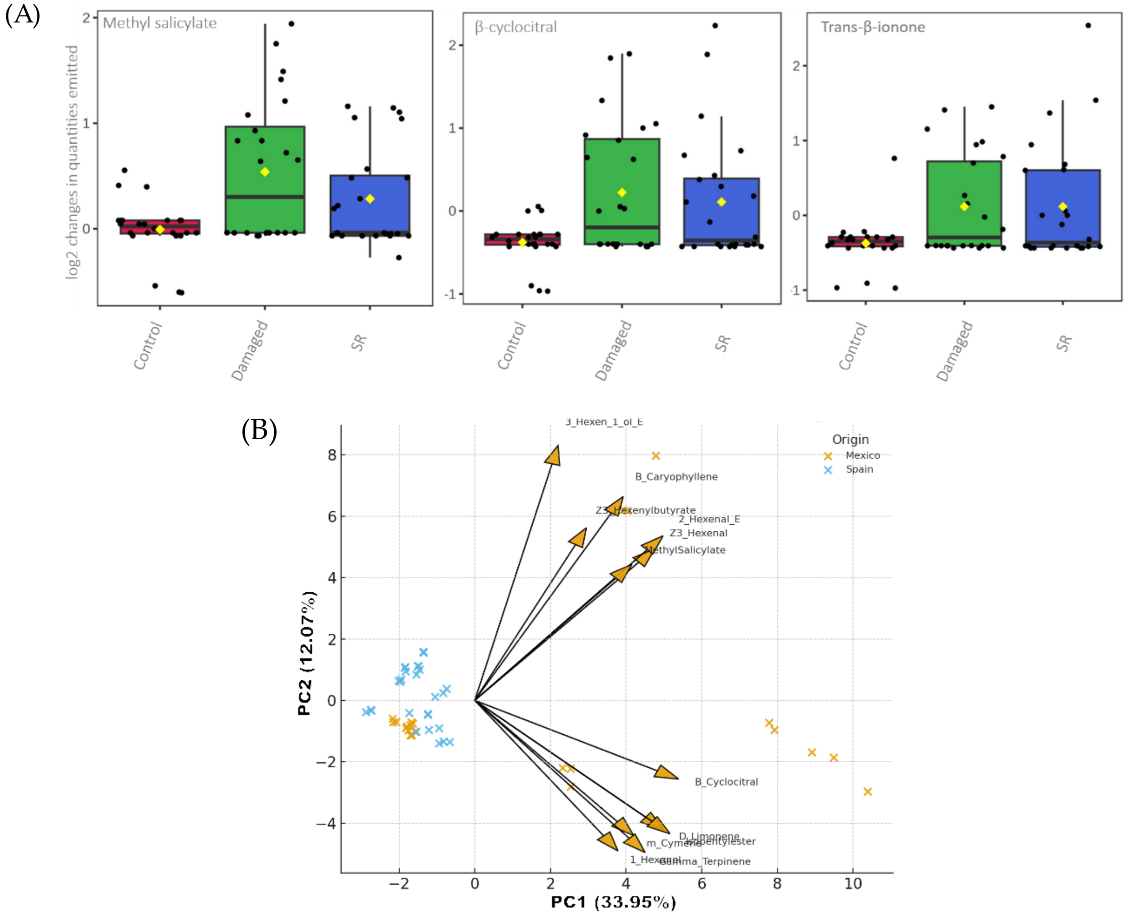

Figure S3. (A) Relative quantification of volatile compounds methyl salicylate, β-cyclocitral, and trans-β-ionone in *Datura stramonium* populations revealed significant differences, with increased emission observed in the damage treatment, followed by the systemic resistance (SR) treatment. Data were transformed into natural logarithms. Differences were confirmed by Tukey's post-hoc tests ($p < 0.05$). (B) Principal Component Analysis (PCA) biplot showing the separation of samples according to Origin and the type of associated metabolites (the metabolite loading vectors). Each point represents one sample, and arrows indicate the contribution and direction of the most influential metabolites driving the separation along PC1 and PC2.

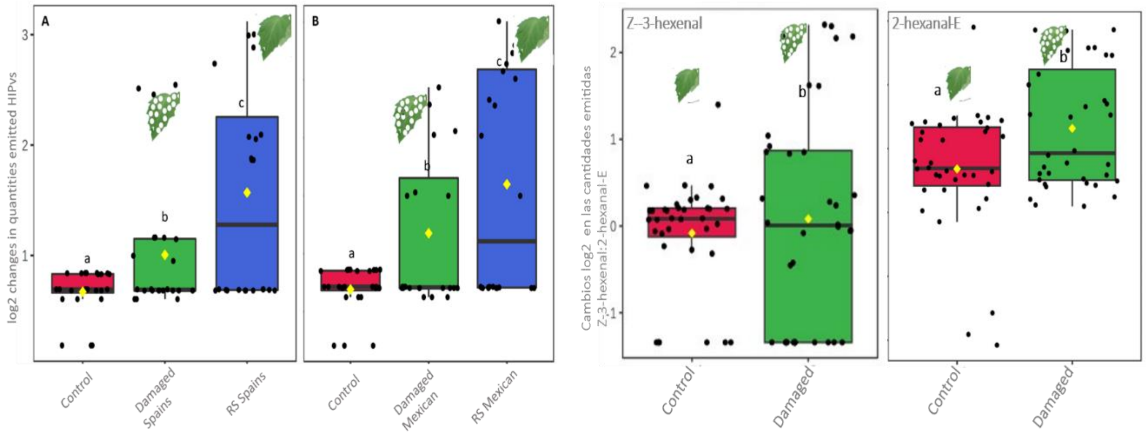

Figure S4. Differential VOC Response to leaf damage in *Datura stramonium* populations. Bar plots A and B quantify HIPVs changes in Spanish and Mexican plant populations, respectively, under control, damage, and SR treatments. Different letter indicate significant differences (Tukey test $p < 0.05$). Boxplots detail ratio of Z-3-hexenal:2-hexenal-E concentrations control and damaged, showing a decrease in the metabolite Z-3-hexenal and an increase in 2-hexanal-E. in the treatment of damage.

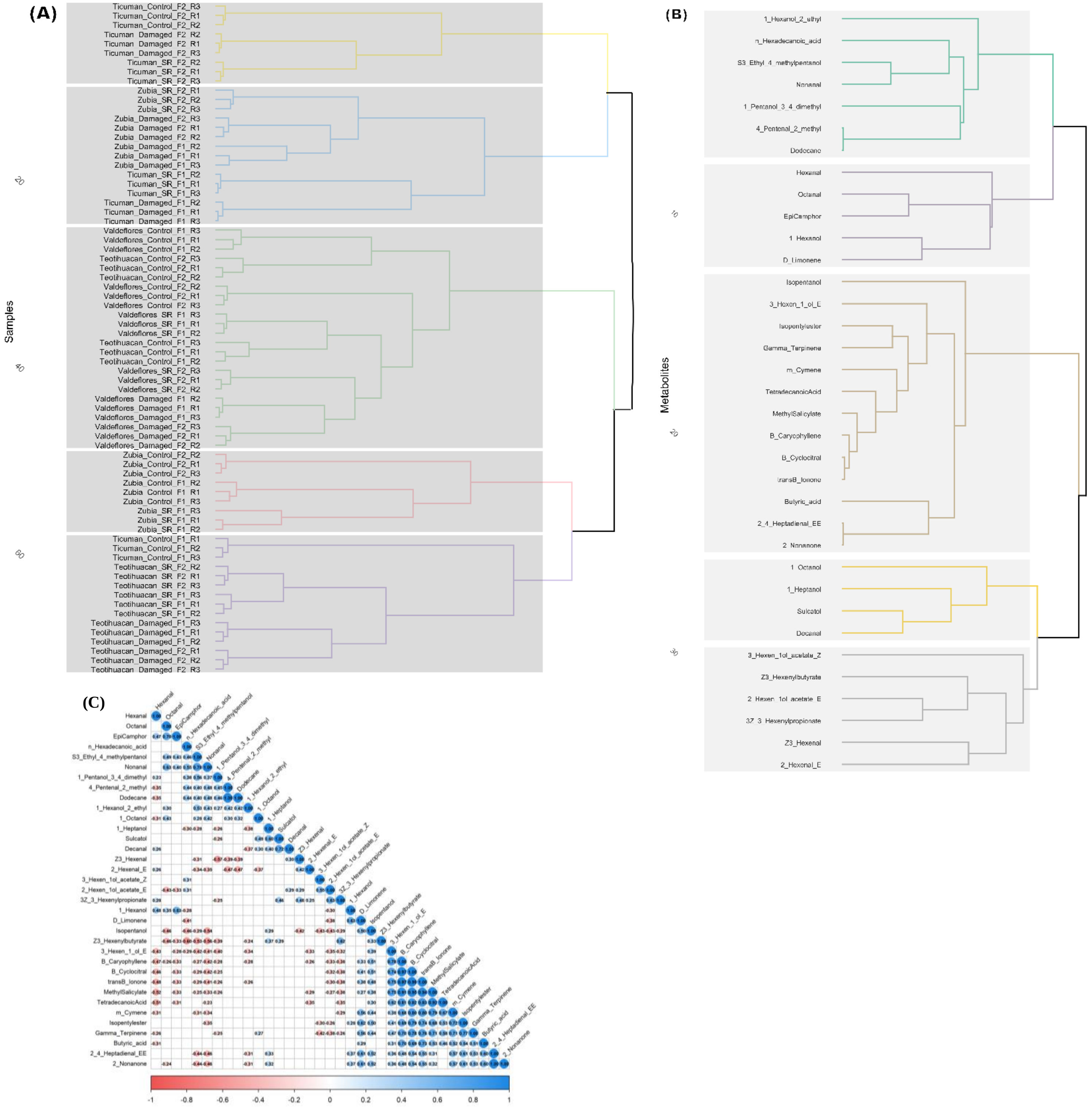

Figure S5. Hierarchical clustering and Spearman correlations among VOCs data in *D. stramonium*. (A) Dendrogram of samples based on Euclidean distances from Hellinger-transformed VOC intensities; shaded rectangles indicate the k clusters (colors denote cluster membership), labels are formatted as Population_Treatment_Family_Replicate. Axes: Samples (x) and Euclidean distance (y). (B) Dendrogram of metabolites based on 1 − Spearman's ρ across samples; shaded rectangles indicate the k clusters. Axes: Metabolites (x) and 1 − Spearman's ρ (y). (C) Pairwise Spearman correlations of VOCs (Hellinger-transformed), circle color encodes sign (blue = positive, red = negative) and size (diameter) is proportional to $|\rho|$; only BH-FDR significant correlations ($q < 0.05$) are shown, and non-significant matrix cells are white.

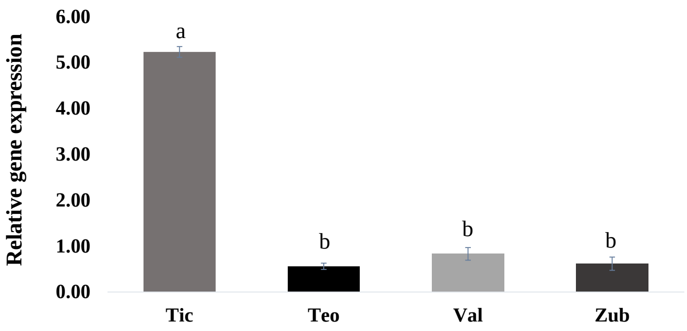

Figure S6. Relative expression profile of *TPS10* of damaged plants of *D. stramonium* as compared to control plants. Bars show mean fold change ($2^{\wedge-\Delta\Delta Ct}$) ± SE relative to Control, measured 18 h after treatment and normalised to *Efα gene*. All data were processed by one-way ANOVA analysis and Tukey-B test. Differences among treatments are indicated by letters ($p \leq 0.05$).

Methods Supplementary: Preliminary studies

Collection of volatile compounds produced by *Datura stramonium*.

Different methods were used to collect the VOCs compounds emitted by plants and capture the highest VOCs richness; the best methods are indicated:

1.-Time to sampling the leaf after mechanical damage (30%):

a) 1 h

b) 10 h

c) 18 h (best option)

d) 24 h

2.-The use of fiber:

a) SPME (DVB/CAR/PDMS), gray color (best option)

b) SPME Fiber Assembly Polydimethylsiloxane/Divinylbenzene (PDMS/DVB), blue color.

3.-How to use it:

a) Direct on the plant, the fiber is placed at a distance not greater than 1 cm from the leaf

b) Use of a plastic bag-leaf-fiber assembly

c) Sterile glass bottle (best option)

d) Collect the compounds from the trichomes of the leaves

4.-How to treat the sample inside the bottle:

a) Plant leaf inside the bottle, without maceration or heat application

b) Plant leaf inside the bottle, with macerations, but without heat application

c) Plant leaf inside the bottle with macerations, and heat application (best option)

5.-Time of heat application to the bottle with the sample and exposure of the fiber:

a) 10 min without fiber plus 15 min with fiber

b) 10 min without fiber plus 30 min with fiber (best option)

c) 10 min without fiber plus 60 min with fiber
